# Ligand-dependent kinase activity of MERTK drives efferocytosis in human iPSC-derived macrophages

**DOI:** 10.1038/s41419-021-03770-0

**Published:** 2021-05-25

**Authors:** Florian Wanke, Simon Gutbier, Anna Rümmelin, Malte Steinberg, Lindsey D. Hughes, Mascha Koenen, Juliana Komuczki, Daniel Regan-Komito, Sagie Wagage, Julia Hesselmann, Ralf Thoma, Doris Brugger, Tony Christopeit, Hayian Wang, Floriane Point, Remy Hallet, Sourav Ghosh, Carla V. Rothlin, Christoph Patsch, Barbara Geering

**Affiliations:** 1 Immunology, Infectious Diseases and Ophthalmology (I2O) Discovery and Translational Area, Roche Innovation Center, Basel, Switzerland; 2 Roche Pharma Research and Early Development, Therapeutic Modalities, Roche Innovation Center, Basel, Switzerland; 3 Yale School of Medicine, Department for Immunobiology, Yale University, New Haven, Connecticut USA; 4 Roche Pharma Research and Early Development, Roche Innovation Center München, Penzberg, Germany; 5 Yale School of Medicine, Department for Neurology, Yale University, New Haven, Connecticut USA; 6 Yale School of Medicine, Department for Pharmacology, Yale University, New Haven, Connecticut USA; 7 Present Address: Laboratory of Molecular Metabolism, Rockefeller University, New York, New York USA; 8 Present Address: BlueRock Therapeutics, New York, New York USA

**Keywords:** Immune cell death, Peritoneal macrophages

## Abstract

Removal of apoptotic cells by phagocytes (also called efferocytosis) is a crucial process for tissue homeostasis. Professional phagocytes express a plethora of surface receptors enabling them to sense and engulf apoptotic cells, thus avoiding persistence of dead cells and cellular debris and their consequent effects. Dysregulation of efferocytosis is thought to lead to secondary necrosis and associated inflammation and immune activation. Efferocytosis in primarily murine macrophages and dendritic cells has been shown to require TAM RTKs, with MERTK and AXL being critical for clearance of apoptotic cells. The functional role of human orthologs, especially the exact contribution of each individual receptor is less well studied. Here we show that human macrophages differentiated in vitro from iPSC-derived precursor cells express both AXL and MERTK and engulf apoptotic cells. TAM RTK agonism by the natural ligand growth-arrest specific 6 (GAS6) significantly enhanced such efferocytosis. Using a newly-developed mouse model of kinase-dead MERTK, we demonstrate that MERTK kinase activity is essential for efferocytosis in peritoneal macrophages in vivo. Moreover, human iPSC-derived macrophages treated in vitro with blocking antibodies or small molecule inhibitors recapitulated this observation. Hence, our results highlight a conserved MERTK function between mice and humans, and the critical role of its kinase activity in homeostatic efferocytosis.

## Introduction

Apoptosis is a central process in the development and homeostasis of multicellular organisms. This specific form of cell death is most commonly induced in non-immunogenic conditions and its disposal is usually immunologically silent. Defects in efferocytosis may cause accumulation of apoptotic cells that subsequently undergo secondary necrosis^1–5^. This is thought to result in the activation of innate immune cells, e.g., plasmacytoid dendritic cells^6^ and macrophages^7^ to produce pro-inflammatory cytokines and thus enhance local inflammation or even manifest as systemic inflammation. Different autoimmune diseases have been linked to defects in efferocytosis^1^, including systemic lupus erythemathosus (SLE)^4,5^.

In the broad repertoire of phagocytic receptors expressed on macrophages and dendritic cells, including many that recognize opsonized phosphatidylserine (PtdSer), the TAM family of receptor tyrosine kinases (RTKs) has been demonstrated to be highly relevant for the clearance of apoptotic cells^8,9^. TAM RTKs consist of TYRO3 (also known as BYK, DTK, RSE, SKY, and TIF), AXL (also known as UFO) and MERTK (also known as MER) with some of the family members being expressed by not only macrophages and dendritic cells^10–14^ but also by Sertoli cells in the testis^15,16^ and by retinal pigment epithelial (RPE) cells in the eye^17,18^. These receptors function as indirect sensors for apoptotic cells and rely on their natural ligands, growth arrest-specific 6 (GAS6) and protein S (PROS1) to mediate binding to exposed PtdSer and efferocytosis of apoptotic cells^19–23^. Both, GAS6 and PROS1 were shown to bind PtdSer displayed on the outer plasma membrane of apoptotic cells in a calcium dependent manner via gamma-carboxylated glutamic acid residues in the Gla-domain^19–21,23^. GAS6 may bind to all three receptors with varying affinity, while PROS1 binding is restricted to TYRO3 and MERTK^23,24^. However, other PtdSer-independent bridging ligands for TAM RTKs have been described, such as tubby, tubby-like protein 1, and galectin-3^25,26^. As a result of ligand-mediated binding, apoptotic cells are tethered to the phagocyte, induce TAM RTK signaling and trigger engulfment. Interestingly, mice deficient for TAM RTKs develop spontaneous autoimmunity with elevated levels of dsDNA specific antibodies and pro-inflammatory cytokines resembling the clinical manifestation of SLE^4,14,27–29^. Moreover, *Axl*- and *Mertk*-deficient mice show increased disease activity in experimental models of acute and chronic inflammation^29–32^. In a therapeutic approach, Van den Brand et al. and Gruber et al. could demonstrate that overexpression of TAM RTK ligands reduced the outcome and disease severity in murine models of arthritis and multiple sclerosis^33,34^.

The translatability of these mouse findings and the relevance of AXL and MERTK for efferocytosis in the human system are insufficiently explored.

Therefore, we examined the expression and functionality of individual TAM RTKs, AXL, and MERTK, in human macrophages differentiated in vitro from either induced pluripotent stem cell-derived macrophage progenitors (tissue resident-like macrophages) or from monocytes isolated from human peripheral blood mononuclear cells (infiltrating-like macrophages). Using these in vitro systems, we could demonstrate that biologically active GAS6 enhances efferocytosis in both, iPSC- and monocyte-derived macrophages. Blocking AXL or MERTK by antibodies or small molecule inhibitors in vitro revealed an essential role for MERTK in the phagocytosis of apoptotic cells by human macrophages. In line with these findings, in vivo efferocytosis by macrophages in mice required MERTK, but not AXL function. We show that, under homeostatic conditions, large peritoneal macrophages are the sole cells engulfing apoptotic cells upon intraperitoneal injection in mice. Strikingly, efferocytosis was significantly enhanced by GAS6 administration. In contrast, AXL function was dispensable for macrophage efferocytosis. Using Cas9/CRISPR to edit the endogenous *Mertk* to generate a kinase-inactive MERTK expressing mouse, we demonstrated that MERTK kinase activity was essential for efferocytosis both in vitro and in vivo. Our results highlight the importance of a conserved role of GAS6 and MERTK kinase activity as a key driver of efferocytosis in murine and human macrophages.

## Results

### Human iPSC-derived macrophages express AXL and MERTK

**Fig. 1:**
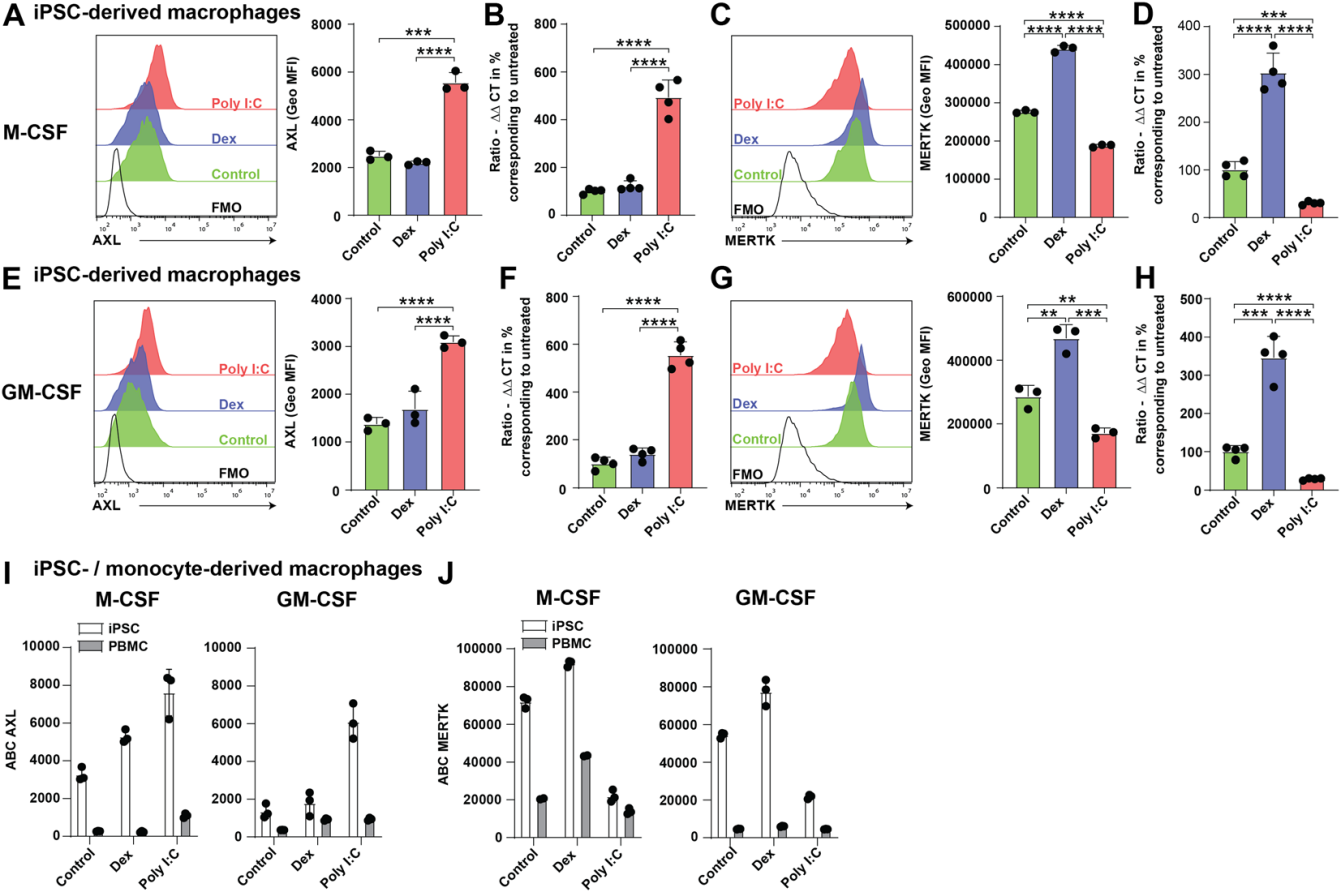
Human iPSC-derived macrophages express AXL and MERTK. **A–H** Macrophage progenitor cells were generated from iPSCs and cultured in vitro using either M-CSF or GM-CSF. Macrophages were stimulated with Poly I:C or dexamethasone or left untreated. After 24 h AXL and MERTK expression was determined by flow cytometry or by qRT-PCR. **A–D** Representative histograms showing expression of AXL and MERTK on M-CSF differentiated macrophages upon stimulation using indicated conditions. Bar graphs show geometrical mean fluorescence intensity of AXL and MERTK or mRNA expression levels of *Axl* and *Mertk*. Shown is mean with SD and individual samples. **E–H** Representative histograms showing expression of AXL and MERTK on GM-CSF differentiated macrophages upon stimulation with indicated conditions. Bar graphs show geometrical mean fluorescence intensity of AXL and MERTK or mRNA expression levels of *Axl* and *Mertk*. Shown is mean with SD and individual samples. **I**, **J** Absolute flow cytometric quantification of AXL and MERTK cell surface expression on iPSC- and monocyte-derived macrophages. Antibodies per cell (ABC) have been determined using a bead based assay. All experiments have been performed with three (flow cytometry) or four (qRT-PCR) technical replicates. Data were representative of at least three individual experiments.

In order to gain insight into the functional role of human TAM RTK orthologues, we employed cellular systems allowing to mimic the generation of infiltrating macrophages (monocyte-derived macrophages) and of tissue resident-like macrophages from iPSC-derived macrophage progenitor cells in vitro^35,36^. Both, human monocyte-derived as well as iPSC-derived macrophages were previously shown to engulf apoptotic cells^36^. In a first step, we used quantification of mRNA levels as well as flow cytometric analysis of cell surface protein amounts to assess expression of *Axl* and *Mertk* in monocyte- or iPSC-derived macrophages under homeostatic, resolving and inflammatory conditions. In contrast to their PBMC-derived counterparts (Supplementary Fig. 1A–D), iPSC-derived macrophages (Fig. 1A–H) expressed detectable protein levels of AXL under all tested conditions. Similar to macrophages differentiated from blood-derived monocytes, AXL expression was upregulated in a comparable manner by Poly (I:C), an analog of double-stranded RNA activating TLR3 (Supplementary Fig. 1A–D, Fig. 1A–H). Treatment with dexamethasone, an immune regulatory glucocorticoid, triggered increased MERTK protein levels in both macrophage preparations. Interestingly, TLR4 signaling by lipopolysaccharide reduced surface distribution of both AXL and MERTK (data not shown), possibly due to receptor shedding by metalloproteases^37^. In order to assess and compare absolute numbers of AXL and MERTK on iPSC- and monocyte-derived macrophages we used a bead based assay. Using this method, we found iPSC-derived macrophages to express ~20 times more MERTK than AXL (~4,000 AXL molecules compared to 80,000 MERTK molecules). Moreover, in line with our findings comparing geometrical mean fluorescence intensity, monocyte-derived macrophages displayed significantly less AXL and MERTK surface receptor density compared to their iPSC-derived counterparts (Fig. 1I–J). In these experiments, an iPS cell clone called SFC840 was used to generate macrophage precursors. In order to verify replicability of these findings in another iPS cell clone, the Neo clone was chosen. Under similar differentiation conditions, AXL and MERTK expression on macrophages was detected with a similar pattern of regulation upon stimulation. However, expression levels of both receptors were slightly lower on macrophages derived from the Neo clone iPSCs (Supplementary Fig. 2B, C).

### GAS6 enhances efferocytosis in vitro in human macrophages

**Fig. 2:**
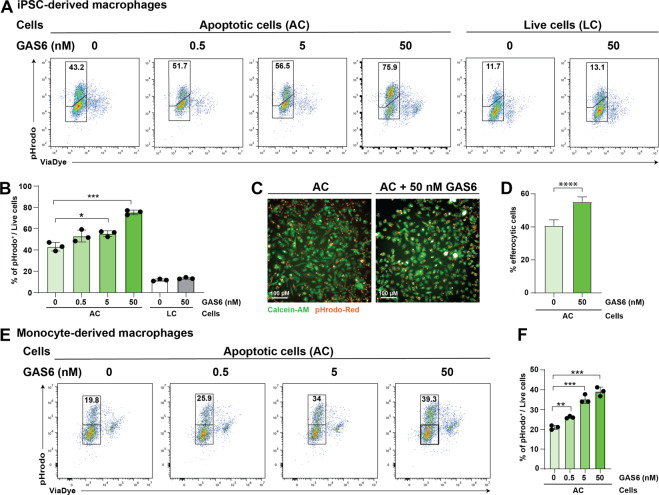
GAS6 enhances efferocytosis in vitro in human iPSC-derived macrophages. **A** Flow cytometric analysis of efferocytosis in vitro using iPSC-derived macrophages. Apoptotic cells (ACs) were generated as described and live cells (LC) were used as control. ACs were labeled with indicated concentrations of GAS6. For LCs, GAS6 was added soluble. Macrophages and ACs were cultured for 2 h at a ratio of 1:6 and analyzed afterwards for pHrodo signal. **B** Frequency of pHrodo^+^ macrophages. Bar graphs show mean with SD and individual samples. **C** High content imaging to determine frequency of efferocytic macrophages. Macrophages were stained with Calcein-AM prior to the assay. ACs were labeled with indicated concentrations of GAS6 and added to macrophages for 2 h. Afterwards, efferocytic macrophages were quantified using the Operetta CLS High-Content Analysis system. **D** Frequency of pHrodo^+^ macrophages. Bar graphs show mean with SD. **E** Flow cytometric analysis of efferocytosis in vitro using monocyte-derived macrophages. **F** Frequency of pHrodo^+^ macrophages. Bar graphs show mean with SD and individual samples. All experiments have been performed with three (flow cytometry) or 32 (imaging) technical replicates. Data were representative of at least three individual experiments.

In a second step, the contribution of TAM RTKs to efferocytosis by human macrophages was evaluated (Fig. 2). To this end recombinant mouse GAS6 was generated in-house, tested for gamma-carboxylation (Supplementary Fig. 3A–F), binding to PtdSer and AXL/MERTK (Supplementary Fig. 3A–F). We also generated recombinant human GAS6, which showed similar gamma-carboxylation and binding characteristics (data not shown), but was not used due to low yields in protein production. To test if serum-derived factors may impact efferocytosis, we differentiated macrophages from iPSC progenitors in the presence or absence of 10% FBS using M-CSF. Moreover, apoptotic cells were washed with EDTA/BSA prior to the assay in order to remove TAM RTK ligands bound to PtdSer^38^. We found efferocytosis in the presence of FBS to be higher compared to macrophages cultured under serum-free conditions (Supplementary Fig. 2A). Strikingly, coating of apoptotic cells with biologically active GAS6 increased the frequency of efferocytic macrophages in a dose-dependent manner in serum-free cultures (Fig. 2A–D). In the presence of serum, GAS6 did not have an additive effect on efferocytosis possibly due to the presence of factors beyond PROS1 (Supplementary Fig. 2A). Enhanced uptake of apoptotic cells by GAS6 was also observed for macrophages differentiated with M-CSF as determined by flow cytometry (Fig. 2A, B) and high content imaging (Fig. 2D, E). As a control, live cells were added and a basal level of uptake by macrophages was detectable in the assay. However, the addition of GAS6 did not have any impact on the engulfment of live cells (Fig. 2A, B). To replicate this assay, we compared macrophages derived from two independent iPS cell clones side by side. Similar results were obtained with macrophages derived from SCF840 or Neo clone of iPS cells (Supplementary Fig. 2C, D). In line with experiments performed in iPSC-derived macrophages, efferocytosis in PBMC-derived macrophages was also found to be significantly enhanced by GAS6 (Fig. 2E, F).

### MERTK kinase activity is required for the clearance of apoptotic cells by murine macrophages in vivo

**Fig. 3:**
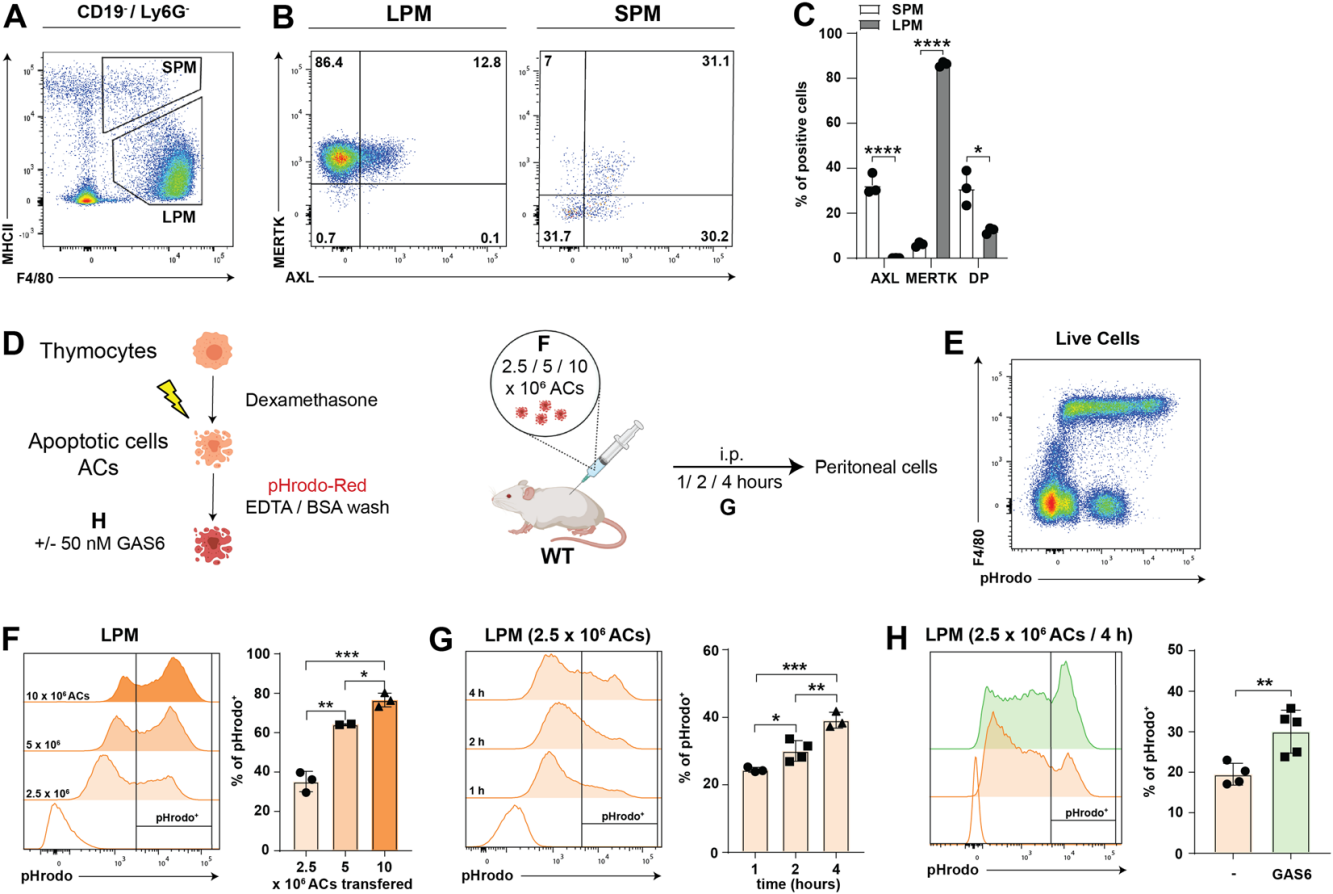
GAS6 augments homeostatic efferocytosis in murine peritoneal macrophages. **A** Gating strategy to identify murine large (LPM) and small (SPM) peritoneal macrophages. Cells were gated as CD19^−^, Ly6G^−^ live cells. **B** Representative flow cytometric analysis for expression of AXL and MERTK on LPM and SPM. **C** Frequency of AXL or MERTK single and double positive LPM and SPM. Bar graphs show mean with SD and individual samples. **D** Experimental set-up to study efferocytosis in the murine peritoneum. Apoptotic cells (AC) were generated by treating murine thymocytes with dexamethasone. Afterwards, cells were labeled with pHrodo-Red and washed with EDTA/BSA and PBS. To set-up the model, initially different numbers of ACs were injected intraperitoneally. Peritoneal exudate cells (PECs) were analyzed at different time points by flow cytometry. Created with BioRender.com **E** Representative flow cytometric analysis of PECs for expression of F4/80 and pHrodo-Red signal. **F** Representative histograms for pHrodo-Red upon injection of different numbers of ACs and PECs isolated after 4 h (Gated on CD19^−^, F4/80^+^ live cells). Statistical analysis of pHrodo^+^ cells. Bar graphs show mean with SD and individual samples. **G** Representative histograms for pHrodo-Red upon injection of 2.5 × 10^6^ ACs and PECs were isolated at indicated time points (Gated on CD19^−^, Ly6G^−^, and F4/80^+^ live cells). Statistical analysis of pHrodo^+^ cells. Bar graphs show mean with SD and individual samples. **H** Representative histograms for pHrodo-Red upon injection of 2.5 × 10^6^ ACs with or without 50 nM GAS6. PECs were isolated at indicated time points (Gated on CD19^−^, Ly6G^−^, and F4/80^+^ live cells). Bar graphs show mean with SD and individual samples. Data were representative of two individual experiments (*n* = 3–5).

**Fig. 4:**
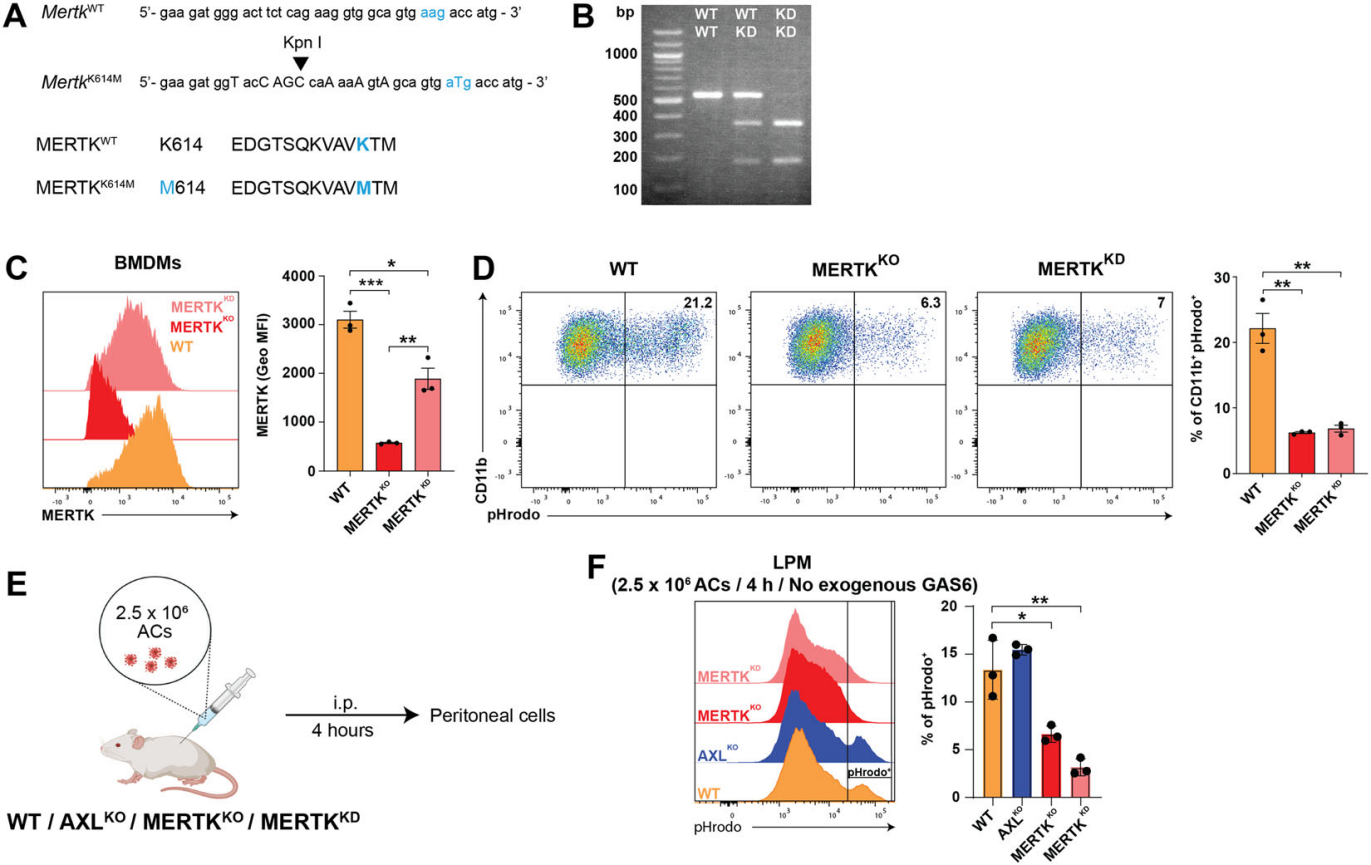
MERTK kinase activity is essential for clearance of apoptotic cells in vivo. **A** (top) targeted codon in *Mertk* for the generation of Mertk^K614M/K614M^ is indicated in blue. A KpnI restriction enzyme site was engineered to aid PCR-based genotyping. (bottom) Amino acid sequence of the targeted region, indicating the introduced mutation at Lysine (K) 614 for Methionine (M) 614. **B** Diagnostic PCR results, after DNA amplification and digestion with KpnI. The WT allele yields one 548 bp product and the mutant allele yields a 184 and a 364 bp band. **C** Representative histograms showing expression of MERTK in bone marrow-derived macrophages from mice with the indicated genotypes. Bar graphs show geometrical mean fluorescence of MERTK. Shown is mean with SEM and individual samples. **D** Representative flow cytometric analysis of efferocytosis with bone marrow-derived macrophages from mice with the indicated genotypes. Macrophages and ACs were co-incubated for 1 h in the presence of serum at a ratio of 1:6 and analyzed afterwards for pHrodo signal. Bar graphs show mean with SEM and individual samples. **E** Experimental set-up to study contribution of AXL and MERTK for efferocytosis in the murine peritoneum. ACs (2.5 × 10^6^) were injected intraperitoneally into mice of indicated genotype. After 4 h PECs were analyzed via flow cytometry. Created with BioRender.com. **F** Representative histograms for pHrodo-Red upon injection of ACs into mice of indicated genotype (Gated on CD19^−^, Ly6G^−^, and F4/80^+^ live cells). Frequency of pHrodo^+^ cells. Bar graphs show mean with SD and individual samples. Data were representative of two individual experiments (*n* = 3–5).

As GAS6 may bind to both AXL and MERTK, we next addressed the relevance of the individual receptors for efferocytosis in vivo. Hence, AXL and MERTK expression on murine peritoneal macrophages was assessed. We found large peritoneal macrophages (LPM, gated as live, CD11c^−^, CD19^−^, Ly6G^−^, CD11b^Hi^, F4/80^Hi^, and MHCII^Lo^) to express MERTK, with some cells to also be positive for AXL (10%). In contrast, the majority of small peritoneal macrophages (SPM, gated as live, CD11c^−^, CD19^−^, Ly6G^−^, CD11b^Hi^, F4/80^Lo^, and MHCII^Hi^) express AXL, but we also detected co-expression with MERTK (Fig. 3A–C). In order to study efferocytosis in vivo, we used a murine model to study efferocytosis in the peritoneum by transferring pHrodo labeled apoptotic thymocytes intraperitoneally into wild-type mice (Fig. 3D). Peritoneal exudate cells were analyzed by flow cytometry to identify efferocytic cells and to monitor the extent of efferocytosis. First, different numbers of apoptotic cells were injected (Fig. 3F) and peritoneal exudate cells subjected to analysis at different time points (Fig. 3G). Interestingly, we found resident large peritoneal macrophages (gated as live, CD11c^−^, CD19^−^, Ly6G^−^, CD11b^Hi^, and F4/80^Hi^) to be the sole cells to take up apoptotic cells (Fig. 3E). Neither dendritic cells nor monocytes or B cells displayed an increase in pHrodo signal. Based on results shown in Fig. 3F, G, we subsequently transferred 2.5 × 10^6^ apoptotic cells and analyzed peritoneal exudate cells after 4 h. We next tested whether the TAM RTK agonist GAS6 augments engulfment of apoptotic cells in this in vivo assay. Recombinant mouse GAS6 was generated in-house, tested for gamma-carboxylation (Supplementary Fig. 2), binding to PtdSer and AXL/MERTK (Supplementary Fig. 3A–F) and administrated along with transfer of apoptotic cells. Strikingly, addition of GAS6 significantly enhanced efferocytosis, as quantified by flow cytometry (Fig. 3H). To expand on these findings, the effect of GAS6 on efferocytosis in vitro was assessed. To this end, macrophages were differentiated from bone marrow precursor cells in the presence of M-CSF. Using pHrodo labeled apoptotic thymocytes, we detected a significant increase in efferocytosis upon addition of GAS6 in bone marrow-derived macrophages (Supplementary Fig. 4A). To determine the contribution of the individual receptors, pHrodo labeled apoptotic cells were injected intraperitoneally into wild-type mice or animals lacking expression of AXL or MERTK (*Axl*^−/−^ or *Mertk*^−/−^ mice). After 4 h, flow cytometric analysis of peritoneal exudate cells revealed comparable uptake of apoptotic cells by wild-type and AXL- deficient peritoneal macrophages. Importantly, a strong reduction in efferocytosis was observed when mice lacked expression of MERTK. This resulted in only 5% of macrophages being pHrodo^Hi^ compared to 14% in wild-type or AXL-deficient macrophages (Fig. 4F). Our observation of MERTK being critical for the uptake of apoptotic cells by mouse macrophages is in line with recent findings using in vitro differentiated murine bone marrow-derived macrophages and ex vivo peritoneal macrophages^39^. To further dissect the role of MERTK signal transduction as mediator of efferocytosis, knock-in mice with a mutation in the ATP coordinating Lysine (K^614^) to Methionine at the *Mertk* locus were generated (MERTK^KD^ mice, Fig. 4A–D). Briefly, a homology-directed repair oligo, containing 63 bp of flanking DNA on each side of the desired mutation, was designed. The guide sequence also incorporated silent mutations to prevent recutting by the Cas9 enzyme, after repair. A restriction enzyme binding site (KpnI) was introduced in the repaired reading frame to allow for the identification of *MERTK*^KD^ mice by PCR (Fig. 4A, B). BMDM from *MERTK*^KD^ expressed significant amounts of MERTK on their surface in comparison to *MERTK*^KO^ BMDMs, indicating that the engineered mutation did not impair MERTK expression (Fig. 4C). However, the expression of MERTK on the surface of *MERTK*^KD^ BMDMs was lower than in BMDM from WT mice (Fig. 4C). Notwithstanding, we found macrophages from MERTK^KD^ mice to exhibit a similar reduction in the uptake of apoptotic cells as macrophages isolated form MERTK KO animals (Fig. 4F). This finding indicates that effective efferocytosis in peritoneal macrophages in vivo requires MERTK as a trigger of downstream signaling via its kinase activity.

### MERTK kinase activity is critical for efferocytosis in human macrophages in vitro

**Fig. 5:**
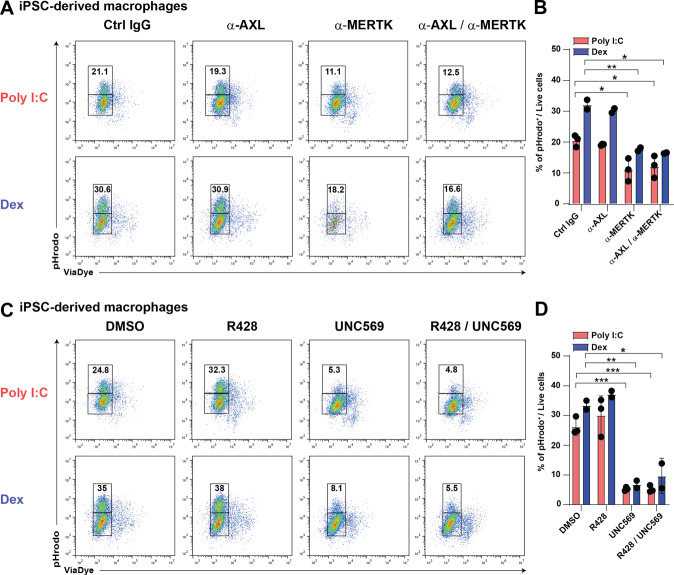
MERTK kinase activity is critical for efferocytosis in human iPSC-derived macrophages in vitro. **A** Flow cytometric analysis of efferocytosis in vitro using iPSC-derived macrophages. Macrophages were stimulated with Poly (I:C) or dexamethasone for 24 h prior to the assay. ACs were generated as described and labeled with 50 nM GAS6. Prior to addition of apoptotic cells, macrophages were incubated with indicated antibodies for 30 min. **B** Frequency of pHrodo^+^ macrophages. Bar graphs show mean with SD and individual samples. **C** Flow cytometric analysis of efferocytosis in vitro using iPSC-derived macrophages. Macrophages were stimulated with Poly (I:C) or dexamethasone for 24 h prior to the assay. ACs were generated as described and labeled with 50 nM GAS6. Prior to addition of apoptotic cells, macrophages were incubated with indicated inhibitors for 30 min. **D** Frequency of pHrodo^+^ macrophages. Bar graphs show mean with SD and individual samples. Data were representative of at least three individual experiments with technical replicates.

In order to dissect the contribution of AXL versus MERTK in tissue resident-like macrophages in vitro, we used two experimental approaches. On the one hand, we blocked binding of apoptotic cells to AXL or MERTK using antibodies. Upon addition of MERTK antibodies, the overall rate of efferocytosis in vitro was significantly reduced, whereas blockade of AXL had no effect (Fig. 5A, B). Moreover, blockade of both receptors simultaneously did not have an additive effect compared to blockade of MERTK only (Fig. 5A, B). Neither upregulation of MERTK by dexamethasone treatment nor upregulation of AXL by Poly (I:C) stimulation impacted on this finding (Fig. 5A, B). These observations are in line with our in vivo experiments in mice lacking AXL, MERTK, or both receptors. Next, we used the small molecule inhibitors R428 (AXL inhibitor) or UNC569 (MERTK inhibitor) to determine the importance of signal transduction downstream of AXL or MERTK in efferocytosis. In line with observations in vivo using MERTK kinase-dead mice (Fig. 5D, E), inhibition of MERTK signaling almost completely abolished the uptake of apoptotic cells (Fig. 5C, D). Both inhibitors were used at a concentration of 20 nM at which they have been described not to inhibit other TAM RTK family members. Hence, our findings indicate that MERTK expression and kinase activity are crucial for efferocytosis in human tissue-resident macrophages.

## Discussion

Several receptors have been implicated in the uptake of apoptotic cells by different phagocytes. However, TAM RTKs have been demonstrated to be highly relevant during this process in dendritic cells and macrophages^39–41^. As an increasing diversity of macrophages and dendritic cells are being described, and these myeloid cells fulfill important immunological functions, we studied the individual contribution of AXL and MERTK in efferocytosis in vivo and moreover, were able to recapitulate our findings using in vitro differentiated human macrophages^38,42,43^. In the murine peritoneum we found solely resident macrophages to engulf apoptotic cells with surprisingly no detectable efferocytosis in dendritic cells or other immune cells in our experiments. In contrast, Subramanian et al. have used a similar model and found splenic dendritic cells to engulf apoptotic cells which was dependent on AXL expression^40^. However, dendritic cells represent only a minor population in the peritoneum and furthermore migrate to adjacent lymphoid organs upon stimulation or uptake of cargo. Moreover, in their work, Subramanian et al. injected apoptotic cells intravenously in contrast to our experiments. Splenic dendritic cells sample for blood borne antigens due to their localization in the tissue and therefore may exhibit stronger phagocytic capacity using this administration route of apoptotic cells.

For peritoneal macrophages we could demonstrate that GAS6 enhances efferocytosis in vivo and in vitro. Protein S is known to be highly abundant in serum, therefore we performed our assays in serum containing medium and found efferocytic capacity of macrophages to be significantly elevated compared to those cultured under serum-free conditions. Importantly, opsonization of apoptotic cells with GAS6 did not further increase their uptake in the presence of serum which may be due to Protein S already maximally occupying apoptotic cell exposed PtdSer. In contrast to Protein S, GAS6 has been shown to bind to all three TAM RTK family members^23,24^ but we did not detect significant levels of TYRO3 in our in vitro differentiated human macrophages and focused on the functionality and relevance of AXL and MERTK.

In both, murine and human, macrophages we found MERTK expression and kinase activity to be essential for efferocytosis. Our findings hold true for efferocytosis in murine peritoneal macrophages in vivo as well as human macrophages in vitro. Using kinase inhibitors for MERTK or MERTK kinase dead mice, we found signal transduction via MERTK to be essential in this process. The overall reduction in efferocytosis in vitro was even more pronounced if MERTK kinase activity was blocked compared to blockade of the receptor by antibodies. Although we have used small molecule inhibitors to block MERTK at low concentrations we cannot rule out the possibility of other relevant kinases being blocked by the treatment, thereby resulting in pronounced reduction in efferocytosis. However, given the similar reduction of efferocytosis in mice with specific mutation in the MERTK kinase domain this effect is most likely due to high relevance of MERTK over other receptors. In contrast, AXL seemed not be involved in the uptake of apoptotic cells in the experiments performed for this study. Current literature suggests that AXL is mainly expressed and relevant during inflammation^38,41^. This is partially reflected by the strong increase of AXL protein levels upon stimulation with Poly (I:C) and type I interferons in human macrophages. In addition, it has been demonstrated that inflammatory signaling may trigger the cleavage of MERTK via ADAM metalloproteases thereby limiting its pro-resolving functions^13,30,37,44^. Hence, a minor role for AXL in efferocytosis by macrophages upon Poly (I:C) treatment was demonstrated^38^, which could not be confirmed in this study. Potential explanations for these differences may be assay conditions or cell types used. Compared to iPSC-derived macrophages, cells differentiated from PBMC-derived monocytes were found to express lower levels of TAM RTKs, especially with regards to AXL^36^. Importantly, albeit AXL expression is increased upon Poly (I:C) treatment of macrophages, surface protein levels are still much lower than for MERTK (Fig. 1I, J). Further studies are needed to analyze the role of AXL expression and functionality on macrophagesduring inflammation. For example, in vivo experiments). Further studies are needed to analyze the role of AXL expression and functionality on macrophages during inflammation. For example, in vivo experiments would be of interest wherein induction of peritoneal inflammation triggers recruitment of monocytes and replacement of resident peritoneal macrophages. Furthermore, the functional role of TYRO3 and AXL have mainly been attributed to the regulation of cytokine secretion and activation in dendritic cells, a cell type we did not investigate in vitro.

In general, TAM RTKs represent a promising target in autoimmunity as agonistic triggering of these receptors by overexpression of GAS6 has been shown to decrease disease severity in murine models of rheumatoid arthritis and multiple sclerosis^33,34^. Our study highlights the relevance of TAM RTKs for efferocytosis to be well conserved among species as documented by the reproducibility of our findings in murine and human macrophages. This is of particular interest, as polymorphisms in the *Mertk* gene were found to be associated with human autoimmune diseases. Therefore, studying TAM RTK expression and functionality in more detail, especially during inflammation may shed light on their relevance in disease pathology and potential druggability.

## Material and methods

### Cloning of murine *gas6* and generation of a polyclonal cell line

The gene for murine gas6 (Uniprot: Q61592) was codon optimized for HEK293 cells and synthesized by Genscript. At the 5′ part a Kozak sequence (GCCGCCACC) was introduced and the natural signal peptide (1–27) was replaced by an Ig-kappa signal peptide (METDTLLLWVLLLWVPGSTGDAAQPA). At the C-terminus a Thrombin-Avi-8xHis-EPEA tag was used (GGR LVPRGS GLNDIFEAQKIEWHESHHHHHHHH EPEA). A NotI site was introduced between the tag and gene as a linker (GGR) sequence with an additional Guanosine as base pair (GGCGGCCGC) to be in frame. The gene was cloned as a NheI/ XhoI fragment into the pcDNA3.1(+) vector resulting in pcDNA3.1(+)-NheI-KOZAK-IgK-mGAS6(28–674)-Thr-Avi-8xHis-EPEA-XhoI. HEK293F cells were transfected with this vector at 1.22 × 10^6^ cells/ml, Viability of 97.5% in a 125 ml Corning flask with Free-style medium. Pools of transfectants were selected by addition of 0.5 mg/ml G418 after 48 h. After 6 days 3% fetal calf serum (FCS) (Sigma, Cat # F4135) was added. After additional 12 days to FCS was reduced to 0.5%. After a total of 24 days, the cells were at 93% viability and protein expression was performed.

### Protein expression and purification

Recombinant full-length murine GAS6 was expressed using HEK293F cells constitutively expressing the mGAS6_Thr-Avi-8His-EPEA. Recombinant cell line was freshly thawed and expanded. Expression was started at a cell number of 0.47 × 10^6^ and a viabilty of 94% in five Fernbach flasks (3 L), each containing 1 L of medium. Free Style Medium was used with 1 mg/ml G418 and 10 µg/ml Vitamin K1 (Konakion ROCHE). Supernatant was harvested after 72 h by centrifugation at 10000 × *g* in 0.5 tubes for 30 min at 4 °C. Afterwards, supernatant was supplemented with one bottle of protease inhibitor cOmplete, EDTA-Free (Roche) per 1 L. Supernatant was either stored at −80 °C or used directly for protein purification. All purification steps were performed on ice or at 4 °C on an AektaPure device (GE Healthcare) under endotoxin-free set-up using HPLC-water and cleaning the system from endotoxin. Nickelsulfat was added to a final concentration of 0.5 mM to the supernatant. The pH was adjusted to 7.0 and the supernatant filtered through a Steritop 45 mm Neck Size Millipore Express PLUS 0.22 µm PES (Millipore) prior to chromatography on a Ni^2+^-IMAC HisTrap HP 5 ml column (GE Healthcare). The column was equilibrated with buffer A consisting of 50 mM Hepes/NaOH (pH 7.0), 300 mM NaCl, 5% glycerol, 0.35% CHAPS (PanReac Applichem), 10 mM Imidazole, 1 mM CaCl_2,_ 6 tablets per liter protease inhibitor cOmplete, EDTA-Free (Roche). The culture supernatant was passed with at 5 ml/min overnight over the column. After washing with 10 volumes of the same buffer,followed by a stepwise elution of 20, 40, 50 (6 CV/step), and a gradient to 500 mM imidazole was applied in the same buffer over 15 CV. The fractions containing murine GAS6 were pooled and desalted by passing over a HiPrep 26/10 Desalting 53 ml (GE Healthcare 17-5087-01) with a flow of 10 ml/min in several runs equilibrated with 20 mM Bis TRIS Propane (pH 7.0), 5% glycerol, 0.35% CHAPS (PanReac Applichem), 1 mM CaCl_2,_ 4 tablets per liter protease inhibitor cOmplete, EDTA-Free (Roche). Fractions containing murine GAS6 were pooled, centrifuged (30,000x*g* for 30 min) and loaded on an anion exchanger HiTrap Q 1 ml (GE Healthcare 17-1153-01) equilibrated with the same buffer as the desalting column and eluted by a gradient to 1 M KCL over 25 CV. Fractions were analyzed by SDS-PAGE, Western blot, and RP-HPLC. Fractions containing murine GAS6 with gamma-carboxylation were pooled and dialyzed in a cassette (Slide-A-Lyser Dialysis cassette, 10,000 MWCO, 0.5–3 ml) against 20 mM Hepes/NaOH (pH 7.0), 50 mM NaCl, 5% glycerol, 1 mM CaCl_2._ The pool was aliquoted and frozen on dry ice and stored at −80 °C. The overall yield was 1–2 mg of monomeric murine GAS6 with gamma-carboxylated Gla-domain.

### Protein analysis and western blot

Protein analysis and quantification was performed by reverse-phase chromatography using a Poroshell 300SB-C8 2.1 × 75 mm column (Agilent Technologies) operated by an Agilent 1200 HPLC system. Western blot was done with the iBlot from Invitrogen using the iBlot Gel Transfer Stacks Nitrocellulose Mini Kit (Life technologies). Blocking was done using 10% non-fat dry milk (170–6404 Bio-Rad) in PBS-T (0.05% Tween) As primary antibody for detection of gamma-carboxyglutamyl amino acid a murine monoclonal antibody against Gla (3570 Sckisui) was used in a dilution of 1:200. As secondary antibody sheep anti-mouse IgG Horseradish peroxidase (NA913V GE Healthcare was used 1:10,000. All antibodies were diluted in 0.05% Tween-TBS. For detection the ECL prime Western Blotting Detection systems (GE healthcare) was used.

### Surface plasmon resonance (SPR) based interaction studies with murine GAS6

All experiments were performed at 25 °C using a Biacore T200 instrument (Cytiva) and running buffer consisting of 10 mM HEPES, pH 7.4, 150 mM NaCl, and 2 mM CaCl_2_. Lipid membranes were immobilized to an SPR sensor surface using L1 chips (Cytiva). The surface was washed by two 30-second injections of 40 mM *n*-octyl β-d-glucopyranoside (OG) in water, followed by the injection of a lipid solution for 850 s. The surface was stabilized by two 30-second injections of 50 mM NaOH, 1 M NaCl, and blocked by three 30-second injections of 1 mg/ml BSA. A flow rate of 5 µl/min was used in all steps of the immobilization. The lipid solution for the immobilization of membranes containing PtdSer was prepared by dissolving 25 mg of a Porcine Total Brain Extract (Avanti Polar Lipids) in 1 ml of lipid buffer consisting of 10 mM Hepes pH 7.4, 150 mM NaCl, and 30 mM OG. For the control membranes, 5 mg of 1-palmitoyl-2-oleoyl-sn-glycero-3-phosphocholine (POPC) and 5 mg of 1-palmitoyl-2-oleoyl-sn-glycero-3-phosphoethanolamine (POPE) were dissolved in 1 ml lipid buffer. The solutions were placed on a rocker overnight and cleared by centrifugation at 10,000 × *g* for 10 min.For the interaction studies of murine GAS6 with lipid membranes, the protein was injected for 60 s over the surface with the immobilized membranes. The dissociation was followed for 240 s. Prior to the injection, murine GAS6 was diluted in running buffer to a concentration of 320, 160, or 80 nM. To remove bound GAS6 and regenerate the lipid membrane, regeneration buffer consisting of 10 mM Hepes pH 7.4, 150 mM NaCl, and 4 mM EDTA was injected for 60 s. To investigate the Ca^2+^-dependency of the GAS6 interaction with the lipid membranes, the interaction studies were repeated using the regeneration buffer as running buffer. The interaction studies of murine GAS6 with human MERTK and human AXL were carried out at the surface of lipid membranes. At first, the membranes were loaded with GAS6 by a 120-second injection of 120 nM GAS6 over an SPR sensor surface with immobilized membranes. The injection was directly followed by a 60-second injection of human recombinant AXL (R&D Systems, Cat.# 154-Al) or MERTK (R&D Systems, Cat.# 891-MR) diluted in running buffer. The dissociation was followed for 240 s. Bound GAS6 was removed and the lipid membranes regenerated by injecting regeneration buffer for 60 s. All sensorgrams were reference and blank subtracted. As a reference, an unmodified surface without lipid membranes was used. Blank injections were carried out using running buffer. A flow rate of 30 µl/min was used for all interaction studies. The data were evaluated using the Biacore T200 evaluation software 3.1 (Cytiva).

### Mice

*Mertk*^−*/*−^ mice were generated by crossing *Mertk*^*fl/fl*^^45^ and *Rosa26*^ERT2^-Cre^+^ (strain 008463, The Jackson laboratory) animals to induce germline deletion. *Axl*^−/−^ mice were generated as previously described^16^. Wild-type control mice were purchased from The Jackson laboratories. All experimental protocols were conducted in accordance with the NIH guidelines and were approved by the Yale Institutional Animal Care and Use Committee. MERTK^KD^ mice were obtained by inducing a mutation in the *Mertk* gene at K^614^ to M^614^ using CRISPR-Cas9 technology. This mutation is known to render MERTK functionally kinase dead. Briefly, a homology-directed repair oligo, containing 63 bp of flanking DNA on each side of the desired mutation, was designed. The guide sequence also incorporates silent mutations to prevent recutting by the Cas9 enzyme after repair. A restriction enzyme binding site (KpnI) was introduced in the repaired reading frame to allow identification of *MERTK*^K614M/K614M^ mice by PCR. The mutation was confirmed by sequencing and genotyping. Following sequences were used:

Transactivating CRISPR RNA (tracerRNA):

GTTTTAGAGCTAGAAATAGCAAGTTAAAATAAGGCTAGTCCGTTATCAACTTGAAAAAGTGGCACCGAGTCGGTGCTTTTTT

Guide Sequence:

AGATGGGACTTCTCAGAAGG

Repair Oligo:

ACTGCTTCATTTCACAGGAGAGTTTGGGTCTGTAATGGAAGGAAATTTGAAGCAAGAAGATGGtACCAGCCAAAAAGTAGCAGTGATGACCATGAAGTGTGAGTTCCTGGCGTGGGACCTGTTCTTCTGGGTGGACAGTCACCTCAATGTC.

### In vivo efferocytosis

To monitor efferocytosis in vivo we performed intraperitoneal transfer of pHrodo labeled apoptotic cells as described elsewhere^46^. In brief, single-cell suspensions of thymi from 3- to 4-week-old mice were prepared noenzymatically in Dulbecco’s phosphate buffered saline with 2% FCS. Apoptotic cells were generated by incubating 5 × 10^6^ thymocytes/ml in culture medium (RPMI1640, 10% FBS, 1% P/S) with 1 μg/ml dexamethasone for 4 h at 37 °C and 5% CO_2_. Afterwards, cells were washed twice with PBS, 1 mM EDTA, 1% BSA and once with PBS, and counted using a hematocytometer. Cells were adjusted to 5 × 10^6^ cell/ml in warm PBS and incubated with 0.1 μg/ml pHrodo-Red (Invitrogen, Carlsbad, CA, USA) for 30 min at RT in the dark, washed with warm PBS, and counted^47^. Different numbers of apoptotic cells were injected in 500 μl sterile PBS intraperitoneally into recipient mice. For some experiments, 50 nM murine recombinant GAS6 (Roche, internally produced) was injected along with apoptotic thymocytes. After 4 h, peritoneal exudate cells were subjected to flow cytometric analysis.

### Flow cytometry of murine peritoneal cells

For flow cytometric analysis, 1 × 10^6^ peritoneal exudate cells were used. Fc-receptors were blocked and cells stained in PBS + 2% FBS for 30 min. Following antibodies were used: CD19-BV421 (115537, Biolegend, San Diego, CA, USA), MHCII-BV510 (107635, Biolegend), CD11c-FITC (117305, Biolegend), Ly6c-PeCy7 (128018, Biolegend), F4/80-APC (123116, BioLegend), and fixable viability dye-APCeF80 (65086514, eBioscience, San Diego, CA, USA). After surface staining, cells were acquired on a FACS LSRII (BD, New Jersey, NJ, USA). Efferocytosis was determined by detection of pHrodo fluorescence. AXL (AF854, R&D, Minneapolis, MN, USA) and MERTK (12–5751–82, eBioscience) antibodies were used to quantify expression by flow cytometry.

### Induced pluripotent stem cell maintenance

All work with human iPSC and the derived cell types was performed under the respective Swiss legislation, ethical guidelines and approval. Following iPSC clones were used in this study: SFC840-03-01 (STBCi026-B, generated in the StemBANCC consortia and deposited at EBiSC) and SBNeo1 (SBAD3-01, reprogrammed with Life Technologies Cytotune Sendai virus). We recently reported an improved and highly scalable variant of the method published by van Wilgenburg et al.^36,48^ for the differentiation of iPSC to primitive macrophages. In brief, for iPSC maintenance culture dishes (Corning) were coated with 12.5 µg/ml rhLaminin-521 (BioLamina). hiPS cells were seeded and cultured in mTesR1 medium (STEMCELL Technologies) at 37 °C with 5% CO_2_ and medium was changed daily. Cells were passaged at 90% confluency.

### Embryoid body generation

To obtain uniformed EBs, iPS cells were detached using Accutase and plated into Aggrewell 800 (STEMCELL Technologies) plates. Therefore, 2 ml mTesR1, supplemented with 10 μM ROCK inhibitor (Y27632, Callbiochem) and containing a single cell suspension of 4 × 10^6^ iPSCs was added to each AggreWell and centrifuged for 3 min at 100 × *g* to assure an even and fast distribution of the iPSC into the AggreWells. The next day, mesoderm and subsequent hemogenic endothelium induction was started by exchange of 75% of the mTeSR1 media with mTeSR1 media supplemented with 50 ng/ml rhBMP4 (biotechne), 50 ng/ml rhVEGF (biotechne), and 20 ng/ml rhSCF (biotechne), and repeated the following 2 days^36^.

### Plating of EBs and continued maturation along the myeloid lineage

At day 4 of differentiation EBs were harvested by gently dislodging the EBs by rinsing the AggreWells with PBS. EBs were collected in a 40 µm strainer and transferred to factory media, consisting of XVIVO15 media (Lonza) supplemented with 2 mM Glutamax, 1% Penicillin/Streptomycin, 50 µg/ml Mercaptoethanol, M-CSF (100 ng/ml), and IL3 (25 ng/ml). EBs were plated with a density of 1 EBs/cm^2^ on cell culture vessels precoated with growth factor reduced Matrigel (354230 Corning). Myeloid factories were further matured as described previously^49^.

### Harvest of macrophage progenitor cells

Macrophage progenitor cells were collected from the supernatant by centrifugation (4 min, 300 g), cells were resuspended and counted. Subsequently differentiation was started.

### Isolation of monocytes from buffy coat

Buffy coats of healthy human donors who gave their informed consent were obtained from the Center for Blood Transfusion, University Medical Center Bern, Switzerland. PBMCs were isolated by Ficoll-Hypaque gradient centrifugation. Monocytes were isolated by using the monocyte isolation kit (negative selection) and the RoboSep device from STEMCELL Technologies (Vancouver, BC, Canada).

### Differentiation of murine bone marrow-derived macrophages (BMDMs)

Bone marrow was collected from femur and tibia of mice, homogenized using a 40 µM cell strainer and red blood cell lysis was performed. Afterwards, cells were differentiated for 7 days in vitro in culture medium (RPMI1640, 20% FBS, and 1% P/S) supplemented with 50 ng/ml murine recombinant M-CSF.

### Differentiation of human macrophages

Macrophage progenitors were differentiated from human iPS cells as described by us and others^35,36,48,49^. A detailed protocol is available in Supplementary Material and Methods. Monocytes or iPSC derived macrophage progenitors were differentiated in X-VIVO 15 medium (2 mM Glutamax, 1% Penicillin-Streptomycin) or for some experiments in RPMI1640 (10% fetal bovine serum, 2 mM Glutamax, and 1% Penicillin-Streptomycin). Cells were plated in 96-well plates with 5 × 10^4^ cells/well in 200 µl medium or in 384-well plates with 4 × 10^3^ cells/well in 50 µl medium. For macrophage differentiation, 100 ng/ml recombinant human GM-CSF or M-CSF (Peprotech, London, UK) were added and cells were cultured at 37 °C with 5% CO_2_. On day four, a 50% medium change with fresh cytokines was performed. For some assays cells were activated for the last 24 h with following stimuli: 10 µg/ml Poly I:C (InvivoGen, San Diego, USA); 1 µM dexamethasone.

### Staining of AXL and MERTK on human macrophages for flow cytometry

Macrophages were differentiated from monocytes or iPSC derived progenitor cells as described above in X-VIVO 15 medium for 6 days. Prior to flow cytometric analysis macrophages were stimulated under indicated conditions for 24 h. Afterward, cells were washed and detached using Accutase according to the manufacturers protocol and transferred into 96-well V-bottom plates. Fc receptors were blocked and cells were incubated in flow cytometry staining buffer (BD) with following antibodies: TYRO-3-FITC (AF854, R&D), AXL-APC (AF154, R&D), MERTK-PE (367608, Biolegend), and fixable viability dye-eFluor780 (eBioscience) for 30 min in the fridge. For quantification of AXL and MERTK the Quantum™ Simply Cellular^®^ Kit from Bangs Laboratories was used, according to the manufacturer protocol. Cells were washed and acquired on a Cytoflex LX flow cytometer (Beckman & Coulter, Brea, CA, USA) and analyzed using FlowJo V10.

### In vitro efferocytosis assay (Flow cytometry)

Macrophages were differentiated in vitro from iPSC derived progenitors or PBMC monocytes as described above. In brief 5 × 10^4^ cells/well were cultured in culture medium (X-VIVO15, 1% P/S, 2 mM Glutamax) with indicated stimuli for seven days in 96-well flat bottom plates. Jurkat cells were maintained in RPMI1640 (2 mM Glutamax, 10% FBS, MEM nonessential aminoacids). Apoptosis was induced by culturing cells at 1 × 10^6^ cells/ml Jurkat medium containing 2.5 µM staurosporine (Sigma) at 37 °C with 5% CO_2_ for 3 h. For live cell controls, Jurkat cells were cultured with addition of solvent (DMSO) under indicated conditions. Afterwards, cells were labeled with pHrodo Red SE (Invitrogen) according to the manufacturer protocol and washed twice with PBS + 1% BSA + 1 mM EDTA) and once with PBS. Apoptosis induction was verified by flow cytometry using the AnnexinV-Fitc staining kit (BD) with fixable viability dye-eFluor780 (eBioscience). For some conditions apoptotic cells were labeled with 50 nM recombinant murine GAS6 (Roche, internally produced) for 15 min in iPSC medium at RT. Apoptotic or live cells were added to macrophages at a ratio of 6:1 (Apoptotic cells: macrophages) in culture medium for 2 h. For some experiments macrophages were incubated with anti-Axl (AF154, R&D), anti-MERTK (AF891, R&D), or control antibody serum (AB-108-C, R&D) at 5 µg/ml for 30 min. Similarly, small molecule inhibitors for AXL (R428, Selleckchem, Zürich, Switzerland) or MERTK (UNC569, Merck, Darmstadt, Germany) or solvent control (DMSO) were added for 30 min to the cells at a concentration of 20 nM. Macrophages were washed once with PBS, detached with Accutase, stained with fixable viability dye in eFluor780 (eBioscience), and analyzed by flow cytometry.

### In vitro efferocytosis assay (Imaging)

Macrophages were differentiated in vitro from iPSC-derived progenitors or PBMC monocytes as described above. In brief 5 × 10^3^ cells/well were cultured in culture medium (X-VIVO15, 1% P/S, and 2 mM Glutamax) with indicated stimuli for 7 days in 384-well flat bottom plates. Prior to addition of apoptotic cells, macrophages were stained with Calcein-AM and DAPI for 30 min in culture medium. Apoptotic or live cells were added to macrophages at a ratio of 6:1 (Apoptotic cells: macrophages) in culture medium for 2 h. For some conditions apoptotic cells were labeled with 50 nM recombinant murine GAS6 (Roche, internally produced). Afterwards, medium was removed and wells were washed twice with PBS ( + / + ) followed by fixation with 4% PFA for 10 min. Plates were washed twice with PBS ( + / + ) and analyzed using the Operetta system (PerkinElmer, Schwerzenbach, Switzerland) with respective software. Macrophages were detected by Calcein staining and quantified for efferocytic cells by pHrodo signal.

### Statistics

Graphs were made by using GraphPad-Prism. Statistical significance was calculated using the unpaired two-tailed *t*-test. Values of *p* < 0.0001, *p* < 0.001, *p* < 0.01, and *p* < 0.05 were marked by four, three, two, and one asterisks, respectively. Data were represented as mean with SD if not stated otherwise.

## Supplementary information

Supplementary Figure 1

Supplementary Figure 2

Supplementary Figure 3

Supplementary Figure 4

Supplementary Figure Legends

## References

[1] Doran A. C., Yurdagul A. & Jr, Tabas I. Efferocytosis in health and disease. *Nat. Rev. Immunol*. **20**, 254–267 (2019). 10.1038/s41577-019-0240-6 PMC7667664 31822793

[2] Kojima Y (2014). Cyclin-dependent kinase inhibitor 2B regulates efferocytosis and atherosclerosis. J. Clin. Invest..

[3] Yurdagul A, Doran AC, Cai B, Fredman G, Tabas IA (2017). Mechanisms and consequences of defective efferocytosis in atherosclerosis. Front. Cardiovasc. Med..

[4] Cohen PL (2002). Delayed apoptotic cell clearance and lupus-like autoimmunity in mice lacking the c-mer membrane tyrosine kinase. J. Exp. Med..

[5] Munoz LE (2010). Autoimmunity and chronic inflammation - two clearance-related steps in the etiopathogenesis of SLE. Autoimmun. Rev..

[6] Lovgren T, Eloranta ML, Bave U, Alm GV, Ronnblom L (2004). Induction of interferon-alpha production in plasmacytoid dendritic cells by immune complexes containing nucleic acid released by necrotic or late apoptotic cells and lupus IgG. Arthritis Rheum..

[7] Hart SP, Alexander KM, Dransfield I (2004). Immune complexes bind preferentially to Fc gamma RIIA (CD32) on apoptotic neutrophils, leading to augmented phagocytosis by macrophages and release of proinflammatory cytokines. J. Immunol..

[8] Thorp E, Cui D, Schrijvers DM, Kuriakose G, Tabas I (2008). Mertk receptor mutation reduces efferocytosis efficiency and promotes apoptotic cell accumulation and plaque necrosis in atherosclerotic lesions of apoe-/- mice. Arterioscler Thromb. Vasc. Biol..

[9] Scott RS (2001). Phagocytosis and clearance of apoptotic cells is mediated by MER. Nature.

[10] Rothlin CV, Ghosh S, Zuniga EI, Oldstone MB, Lemke G (2007). TAM receptors are pleiotropic inhibitors of the innate immune response. Cell.

[11] Cai B (2020). Macrophage MerTK promotes liver fibrosis in nonalcoholic steatohepatitis. Cell Metab..

[12] Camenisch TD, Koller BH, Earp HS, Matsushima GK (1999). A novel receptor tyrosine kinase, Mer, inhibits TNF-alpha production and lipopolysaccharide-induced endotoxic shock. J. Immunol..

[13] DeBerge M (2017). MerTK cleavage on resident cardiac macrophages compromises repair after myocardial ischemia reperfusion Injury. Circ. Res..

[14] Rahman ZS, Shao WH, Khan TN, Zhen Y, Cohen PL (2010). Impaired apoptotic cell clearance in the germinal center by Mer-deficient tingible body macrophages leads to enhanced antibody-forming cell and germinal center responses. J. Immunol..

[15] Chen Y (2009). Functions of TAM RTKs in regulating spermatogenesis and male fertility in mice. Reproduction.

[16] Lu Q (1999). Tyro-3 family receptors are essential regulators of mammalian spermatogenesis. Nature.

[17] Feng W, Yasumura D, Matthes MT, LaVail MM, Vollrath D (2002). Mertk triggers uptake of photoreceptor outer segments during phagocytosis by cultured retinal pigment epithelial cells. J. Biol. Chem..

[18] Prasad D (2006). TAM receptor function in the retinal pigment epithelium. Mol. Cell Neurosci..

[19] Godowski PJ (1995). Reevaluation of the roles of protein S and Gas6 as ligands for the receptor tyrosine kinase Rse/Tyro 3. Cell.

[20] Mark MR, Chen J, Hammonds RG, Sadick M, Godowsk PJ (1996). Characterization of Gas6, a member of the superfamily of G domain-containing proteins, as a ligand for Rse and Axl. J. Biol. Chem..

[21] Nagata K (1996). Identification of the product of growth arrest-specific gene 6 as a common ligand for Axl, Sky, and Mer receptor tyrosine kinases. J. Biol. Chem..

[22] Ohashi K (1995). Stimulation of sky receptor tyrosine kinase by the product of growth arrest-specific gene 6. J. Biol. Chem..

[23] Stitt TN (1995). The anticoagulation factor protein S and its relative, Gas6, are ligands for the Tyro 3/Axl family of receptor tyrosine kinases. Cell.

[24] Lew E. D., et al. Differential TAM receptor-ligand-phospholipid interactions delimit differential TAM bioactivities. *Elife* **3**, e03385 (2014). 10.7554/eLife.03385 PMC4206827 25265470

[25] Caberoy NB, Zhou Y, Li W (2010). Tubby and tubby-like protein 1 are new MerTK ligands for phagocytosis. EMBO J..

[26] Caberoy NB, Alvarado G, Bigcas JL, Li W (2012). Galectin-3 is a new MerTK-specific eat-me signal. J. Cell Physiol..

[27] Lemke G, Rothlin CV (2008). Immunobiology of the TAM receptors. Nat. Rev. Immunol..

[28] Shao WH (2010). Disrupted Mer receptor tyrosine kinase expression leads to enhanced MZ B-cell responses. J. Autoimmun..

[29] Wallet MA (2008). MerTK is required for apoptotic cell-induced T cell tolerance. J. Exp. Med..

[30] Cai B (2016). MerTK cleavage limits proresolving mediator biosynthesis and exacerbates tissue inflammation. Proc. Natl Acad. Sci. USA.

[31] Bosurgi L (2013). Paradoxical role of the proto-oncogene Axl and Mer receptor tyrosine kinases in colon cancer. Proc. Natl Acad. Sci. USA.

[32] Weinger JG (2011). Loss of the receptor tyrosine kinase Axl leads to enhanced inflammation in the CNS and delayed removal of myelin debris during experimental autoimmune encephalomyelitis. J. Neuroinflammation..

[33] van den Brand BT (2013). Therapeutic efficacy of Tyro3, Axl, and Mer tyrosine kinase agonists in collagen-induced arthritis. Arthritis Rheum..

[34] Gruber RC (2014). Targeted GAS6 delivery to the CNS protects axons from damage during experimental autoimmune encephalomyelitis. J. Neurosci..

[35] Takata K (2017). Induced-pluripotent-stem-cell-derived primitive macrophages provide a platform for modeling tissue-resident macrophage differentiation and function. Immunity.

[36] Gutbier S. et al. Large-scale production of human iPSC-derived macrophages for drug screening. *Int. J. Mol. Sci.* **21**, 4808 (2020). 10.3390/ijms21134808 PMC7370446 32645954

[37] Thorp E (2011). Shedding of the Mer tyrosine kinase receptor is mediated by ADAM17 protein through a pathway involving reactive oxygen species, protein kinase Cdelta, and p38 mitogen-activated protein kinase (MAPK). J. Biol. Chem..

[38] Grabiec AM, Goenka A, Fife ME, Fujimori T, Hussell T (2018). Axl and MerTK receptor tyrosine kinases maintain human macrophage efferocytic capacity in the presence of viral triggers. Eur. J. Immunol..

[39] Dransfield I, Zagorska A, Lew ED, Michail K, Lemke G (2015). Mer receptor tyrosine kinase mediates both tethering and phagocytosis of apoptotic cells. Cell Death Dis..

[40] Subramanian M (2014). An AXL/LRP-1/RANBP9 complex mediates DC efferocytosis and antigen cross-presentation in vivo. J. Clin. Invest..

[41] Zagorska A, Traves PG, Lew ED, Dransfield I, Lemke G (2014). Diversification of TAM receptor tyrosine kinase function. Nat. Immunol..

[42] Alivernini S (2020). Distinct synovial tissue macrophage subsets regulate inflammation and remission in rheumatoid arthritis. Nat. Med..

[43] Zizzo G, Hilliard BA, Monestier M, Cohen PL (2012). Efficient clearance of early apoptotic cells by human macrophages requires M2c polarization and MerTK induction. J. Immunol..

[44] Cai B (2017). MerTK receptor cleavage promotes plaque necrosis and defective resolution in atherosclerosis. J. Clin. Invest..

[45] Fourgeaud L (2016). TAM receptors regulate multiple features of microglial physiology. Nature.

[46] Morioka S (2018). Efferocytosis induces a novel SLC program to promote glucose uptake and lactate release. Nature.

[47] Miksa M, Komura H, Wu R, Shah KG, Wang P (2009). A novel method to determine the engulfment of apoptotic cells by macrophages using pHrodo succinimidyl ester. J. Immunol. Methods.

[48] van Wilgenburg B, Browne C, Vowles J, Cowley SA (2013). Efficient, long term production of monocyte-derived macrophages from human pluripotent stem cells under partly-defined and fully-defined conditions. PLoS ONE.

[49] Haenseler W (2017). A highly efficient human pluripotent stem cell microglia model displays a neuronal-co-culture-specific expression profile and inflammatory response. Stem Cell Rep..

