# Supplementary figures and images for: Ligand-dependent kinase activity of MERTK drives efferocytosis in human iPSC-derived macrophages
Source: Cell Death Dis. 2021 May 25;12(6):538. doi: 10.1038/s41419-021-03770-0 (PMC8149813; doi:10.1038/s41419-021-03770-0)

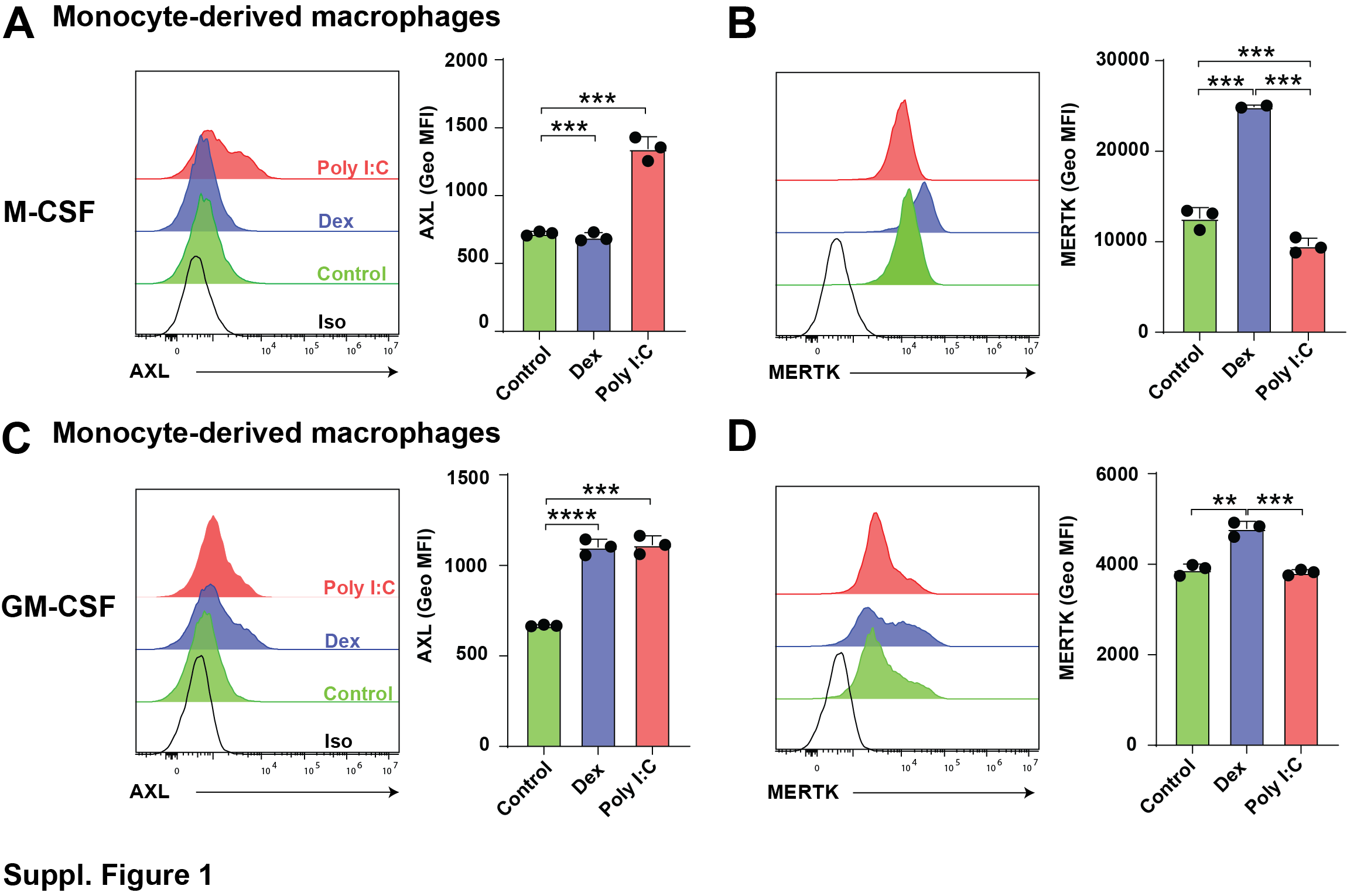

Supplement: Supplementary file 1 — Supplementary Figure 1 [file 41419_2021_3770_MOESM1_ESM.png]

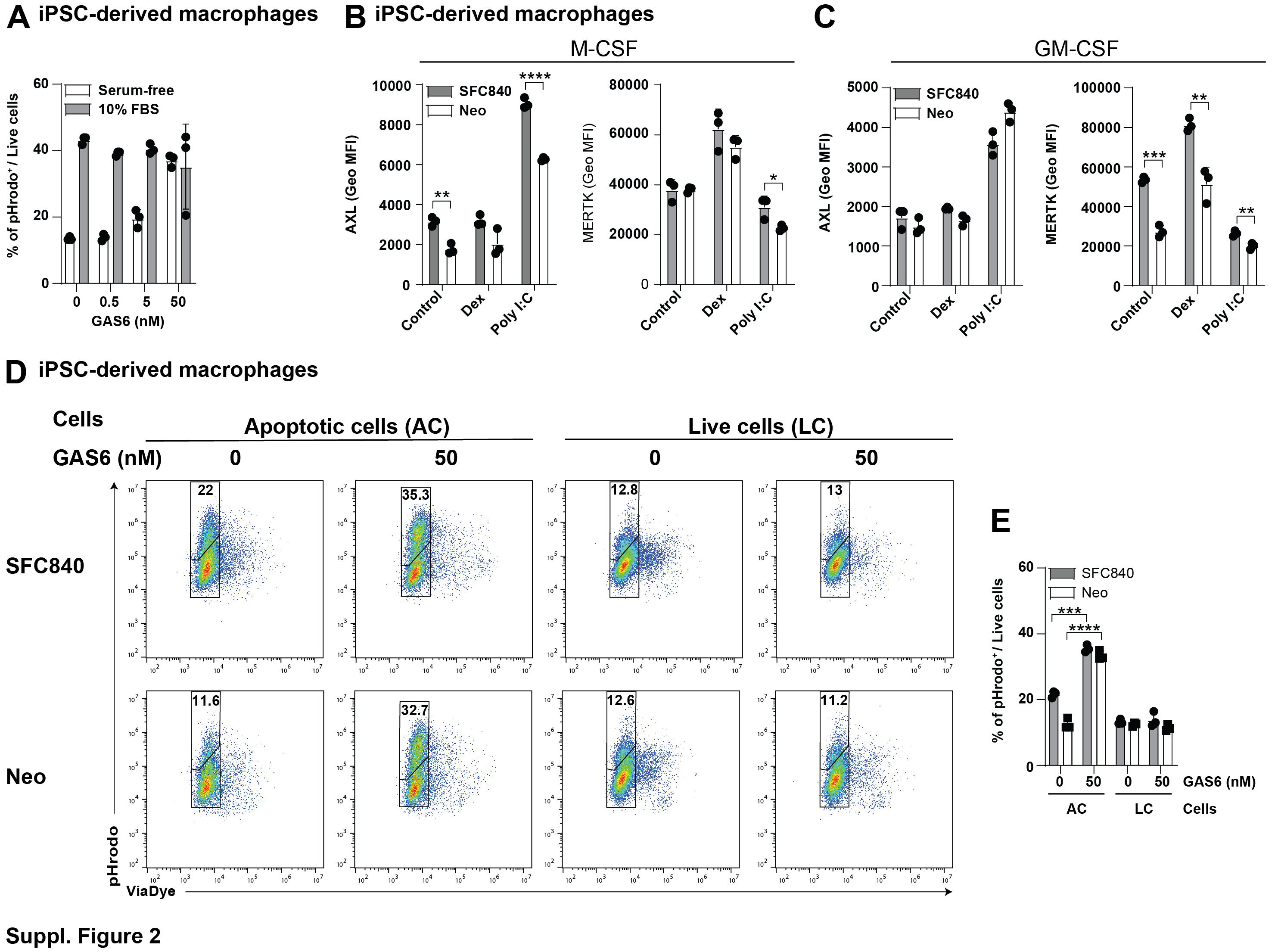

Supplement: Supplementary file 2 — Supplementary Figure 2 [file 41419_2021_3770_MOESM2_ESM.png]

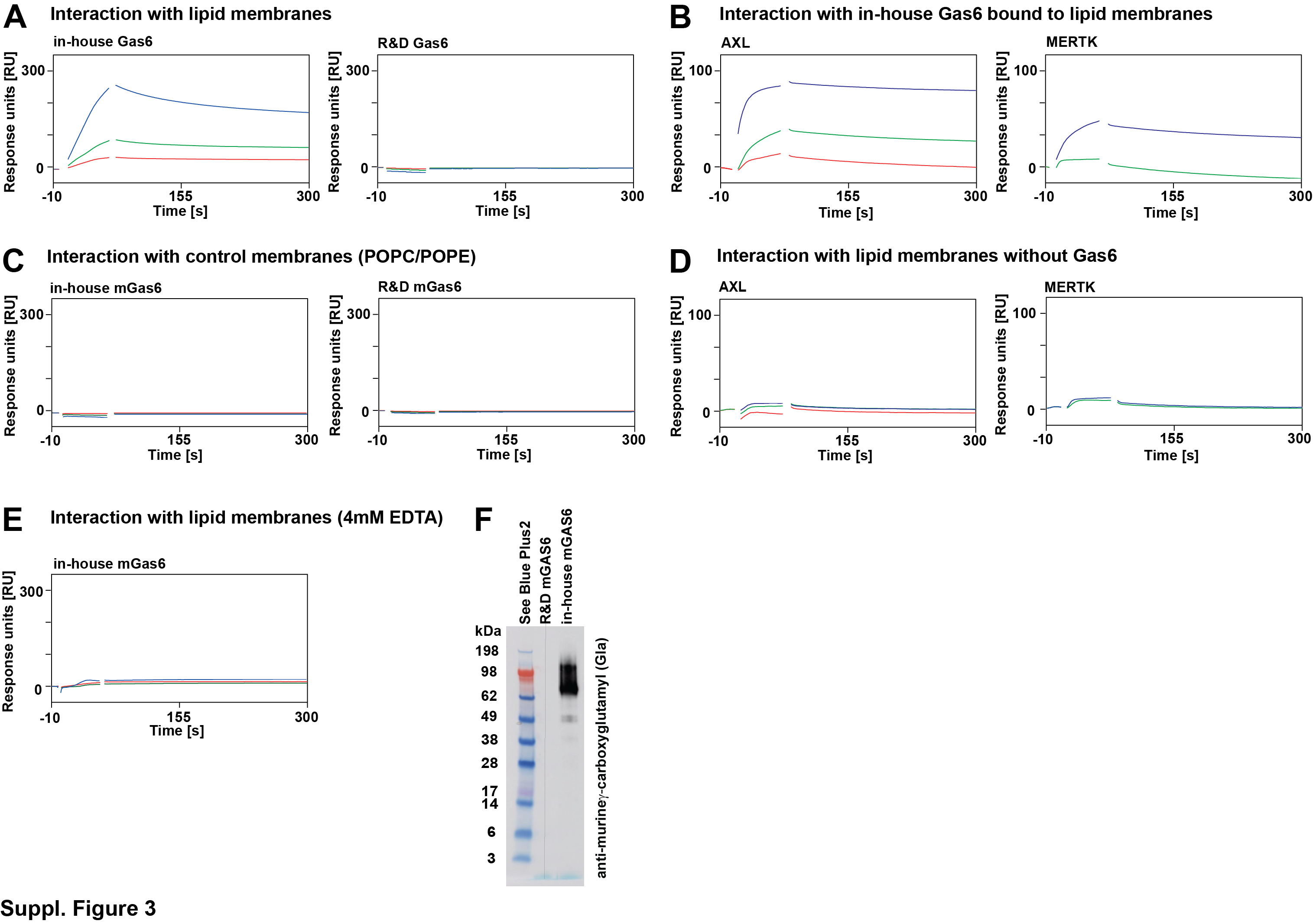

Supplement: Supplementary file 3 — Supplementary Figure 3 [file 41419_2021_3770_MOESM3_ESM.png]

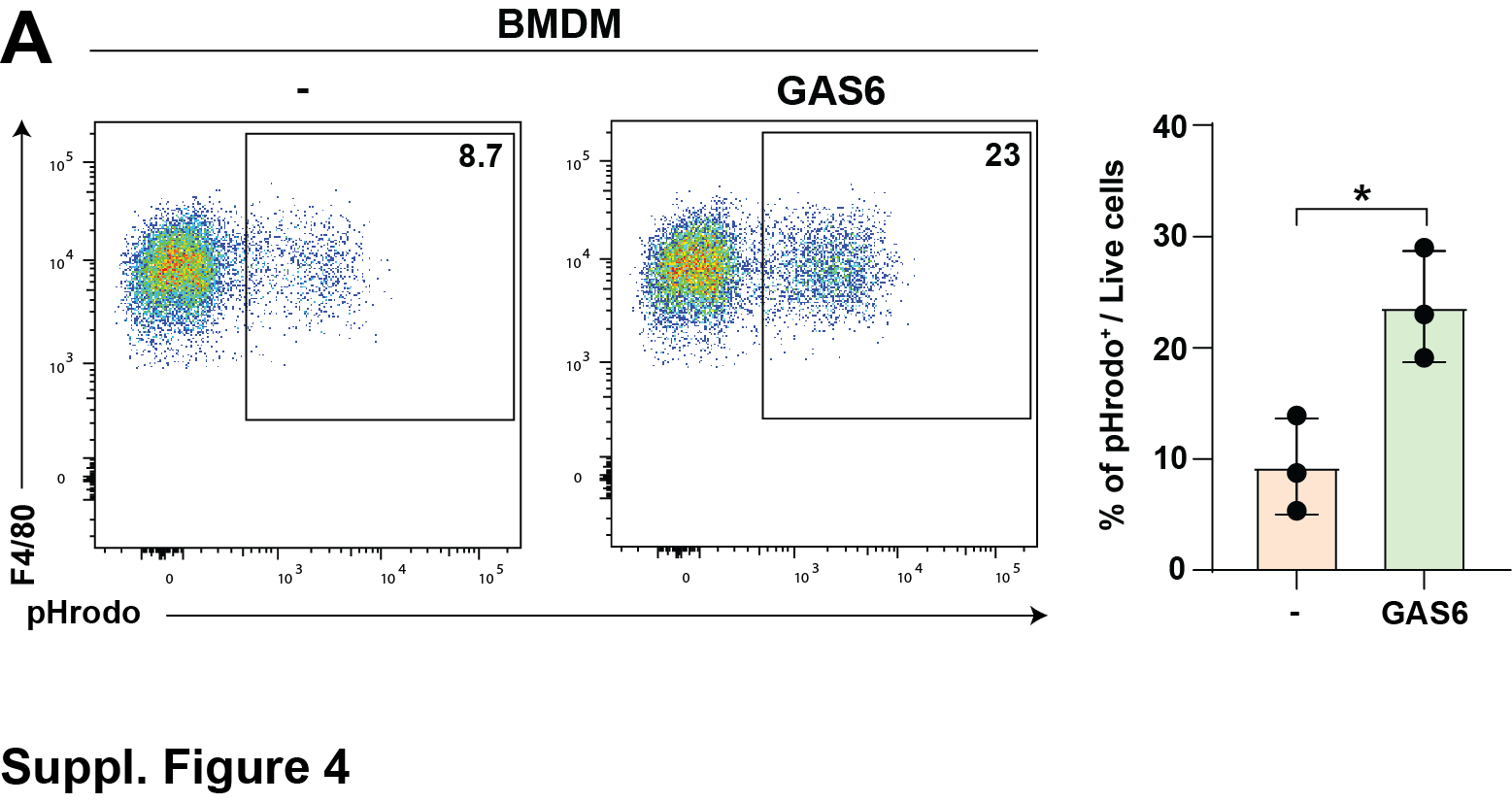

Supplement: Supplementary file 4 — Supplementary Figure 4 [file 41419_2021_3770_MOESM4_ESM.png]
