# Supplementary material for: Ligand-dependent kinase activity of MERTK drives efferocytosis in human iPSC-derived macrophages
Source: Cell Death Dis. 2021 May 25;12(6):538. doi: 10.1038/s41419-021-03770-0 (PMC8149813; doi:10.1038/s41419-021-03770-0)
Supplement: Supplementary file 5 — Supplementary Figure Legends [file 41419_2021_3770_MOESM5_ESM.pdf]

Supplementary Figure Legends

Suppl. Figure 1: Expression of AXL and MERTK in human monocyte-derived macrophages

(A-D) Monocytes were isolated from PBMCs and cultured *in vitro* using either M-CSF or GM-CSF. Macrophages were stimulated with Poly I:C or dexamethasone or left untreated. After 24 hours AXL and MERTK expression was determined by flow cytometry. (A-B) Representative histograms showing expression of AXL and MERTK on M-CSF differentiated macrophages upon stimulation using indicated conditions. Bar graphs show geometrical mean fluorescence intensity of AXL and MERTK. Shown is mean with SD and individual samples. (C-D) Representative histograms showing expression of AXL and MERTK on M-CSF differentiated macrophages upon stimulation using indicated conditions. Bar graphs show geometrical mean fluorescence intensity of AXL and MERTK. Shown is mean with SD and individual samples. Data is representative of at least three individual experiments with three technical replicates.

Suppl. Figure 2: Expression of AXL and MERTK and efferocytosis in iPSC-derived macrophages derived from SFC840 and Neo clone

A) iPSC-derived macrophages were differentiated *in vitro* using serum-containing or serum-free medium. Flow cytometric analysis of efferocytosis under indicated conditions. Bar graphs show mean with SD and individual samples. (B-C) Macrophages were differentiated *in vitro* from macrophage progenitor cells derived from the SFC840 or Neo Clone. For differentiation either M-CSF or GM-CSF was used. Macrophages were stimulated with Poly I:C or dexamethasone or left untreated. After 24 hours AXL and MERTK expression was determined by flow cytometry. Bar graphs show geometrical mean fluorescence intensity of AXL and MERTK. Shown is mean with SD and individual samples (D-E). Macrophages were differentiated *in vitro* from macrophage progenitor cells derived from the SFC840 or Neo Clone. D) Flow cytometric analysis of efferocytosis *in vitro* using iPSC-derived macrophages.

ACs were generated as described and live cells (LC) were used as control. ACs were labeled with indicated concentrations of GAS6. For LCs GAS6 was added soluble. Macrophages and ACs were cultured for 2 hours at a ratio of 1:6 and analyzed afterwards for pHrodo signal. E) Frequency of $pHrodo^{+}$ macrophages. Bar graphs show mean with SD and individual samples. Data is representative of at least three individual experiments with three technical replicates.

Suppl. Figure 3: Generation of biologically active murine GAS6

(A, C, E) Sensorgrams for the interaction of in-house produced murine GAS6 and murine GAS6 from R&D with phosphatidylserine (PtdSer) containing lipid membranes (A) and control lipid membranes without PtdSer (C). GAS6 was injected in three different concentrations (blue: 320 nM / green: 160 nM / red: 80 nM). As a control, the binding of in-house produced GAS6 to PtdSer containing lipid membranes was assessed in the presence of 4 mM EDTA. (B,D) Sensorgrams for the interaction of AXL and MERTK with in-house produced murine GAS6 bound PtdSer containing lipid membranes (B) and to lipid membranes without mGas6 (D). MERTK was injected in two and AXL in three different concentrations (blue: 28 nM / green: 14 nM / red: 7 nM). F) Westernblot analysis for gamma-carboxylation in the Gla-domain of murine recombinant GAS6 produced internally or commercially available from R&D.

Suppl. Figure 4: Efferocytosis in murine bone marrow-derived macrophages

A) Murine macrophages were differentiated from bone marrow precursor cells for 7 days using recombinant murine M-CSF. Apoptotic thymocytes were generated as described, labeled with pHrodo-Red and either left untreated or coated with murine recombinant GAS6 (50 nM). Macrophages and apoptotic thymocytes were co-cultured for 2 hours at a ratio of 1:6. Afterwards, cells were detached and stained for CD11b and F4/80. Efferocytic macrophages were quantified via Flow cytometry. Bar graphs show mean with SD and individual samples of three individual experiments (n=1).
